# COX2 expression is associated with proliferation and tumor extension in vestibular schwannoma but is not influenced by acetylsalicylic acid intake

**DOI:** 10.1186/s40478-019-0760-0

**Published:** 2019-07-11

**Authors:** Felix Behling, Vanessa Ries, Marco Skardelly, Irina Gepfner-Tuma, Martin Schuhmann, Florian-Heinrich Ebner, Ghazaleh Tabatabai, Antje Bornemann, Jens Schittenhelm, Marcos Tatagiba

**Affiliations:** 1 Department of Neurosurgery, Eberhard-Karls University, Hoppe-Seyler Street 3, Tuebingen, Germany; 2 Department of Vascular Neurology, University Hospital Tuebingen, Eberhard Karls University, 72076 Tuebingen, Germany; 3 Eberhard-Karls University, Hoppe-Seyler Street 3, Tuebingen, Germany; 4 Interdisciplinary Division of Neuro-Oncology, Departments of Vascular Neurology and Neurosurgery, University Hospital Tuebingen, Eberhard Karls University, 72076 Tuebingen, Germany; 5 Department of Neuropathology, Eberhard-Karls-University, 72076 Tuebingen, Germany

**Keywords:** Cyclooxygenase 2, Acetylsalicylic acid, Aspirin, Vestibular schwannoma, Acoustic neuroma, Neurofibromatosis, Proliferation, Tumor growth

## Abstract

Acetylsalicylic acid has been linked to a lower risk for different cancer types, presumably through its inhibitory effect on cyclooxygenase 2. This has also been investigated in vestibular schwannomas with promising results suggesting an antiproliferative effect and recently the intake has been recommended for vestibular schwannomas as a conservative treatment option. We constructed tissue microarrays from paraffin-embedded tissue samples of 1048 vestibular schwannomas and analyzed the expression of cyclooxygenase 2 and the proliferation marker MIB1 (Molecular Immunology Borstel) via immunohistochemistry together with clinical data (age, gender, tumor extension, prior radiotherapy, neurofibromatosis type 2, tumor recurrence, cyclooxygenase 2 responsive medication). Univariate analysis showed that cyclooxygenase 2 expression was increased with age, female gender, prior radiotherapy and larger tumor extension. MIB1 expression was also associated with higher cyclooxygenase 2 expression. Schwannomas of neurofibromatosis type 2 patients had lower cyclooxygenase 2 levels. Use of acetylsalicylic acid, non-steroidal anti-inflammatory drugs, glucocorticoids or other immunosuppressants did not show differences in cyclooxygenase 2 or MIB1 expression. Instead, cyclooxygenase 2 expression increases with tumor extension while MIB1 expression is not associated with tumor size. Overall, cyclooxygenase 2 expression is associated with proliferation but not influenced by regular intake of acetylsalicylic acid or other cyclooxygenase 2-responsive medications. Acetylsalicylic acid intake does not alter cyclooxygenase 2 expression and has no antiproliferative effect in vestibular.

## Introduction

Vestibular schwannoma (VS) is a benign tumor entity arising from the vestibular nerve, with an incidence of approximately 1:100,000, accounting for 6–7% of all intracranial tumors [22]. There are no known risk factors for the development of sporadic vestibular schwannomas (sVS), which constitute the majority of cases [4]. Patients suffering from neurofibromatosis type 2 (NF2), an autosomal dominant genetic microdeletion on chromosome 22 resulting in a deficiency of the peptide merlin, typically have bilateral vestibular schwannomas at a relatively young age in addition to other intracranial and intraspinal tumors [8]. In these cases, bevacizumab, a monoclonal antibody targeting vascular endothelial growth factor A (VEGF-A), is an off-label treatment option that can slow tumor growth [23]. No other medical treatment option has been established. Recently a publication recommended to consider aspirin use in patients undergoing observation regarding their vestibular schwannomas [32]. Overall, there is very little evidence for that specific patient subgroup regarding treatment with non-steroidal anti-inflammatory drugs (NSAIDs).

Chronic inflammation has been revealed as a driving mechanism of tumorigenesis in several cancer types. One of the protagonists often discussed is the cyclooxygenase family, a group of enzymes that catalyze the production of prostaglandins, which are crucial for generating and maintaining inflammation [28]. The protective role of NSAIDs for some cancer types has been suspected for several years and was recently confirmed in the NIH-AARP Diet and Health Study [27].

This concept was transferred to vestibular schwannomas a few years ago. Hong et al. demonstrated a proliferative effect of COX2 expression in a group of thirty vestibular schwannomas including fifteen tumors in patients suffering from neurofibromatosis type 2 (NF2) [12]. Later, Dilwali et al. were able to show that the application of acetylsalicylic acid (ASA) decreases COX2 expression and cell growth in vitro, an effect not observed in a normal schwann cell culture [7]. Three retrospective analyses presented conflicting results regarding the antiproliferative effect of ASA intake in sporadic VS. Kandathil et al. demonstrated a correlation of ASA intake and decreased tumor growth [17, 18] while no efficacy was observed in two other cohort studies of similar sample size [13, 21]. A prospective phase II, randomized, double-blind longitudinal study is currently recruiting patients with vestibular schwannomas to investigate the effect of ASA on tumor growth and hearing preservation (NCT03079999).

Overall, the tumorigenic role of COX2 and the possible antiproliferative effect of ASA seems to be a promising area in regard to new therapy options for patients with VS. Most importantly, there is very little data regarding medical therapies in NF2, a patient subgroup that is in need of other treatment options. In this study we assessed the role of COX2 expression in 1048 vestibular schwannomas in regard to clinical data and proliferation.

## Materials and methods

### Study design

The aim of this retrospective analysis was the assessment of the expression of COX2 in vestibular schwannomas with special regard to the correlation with the proliferative marker MIB1 (1) and epidemiological factors (2) as well as the impact of ASA intake on COX2 and MIB1 expression (3).

### Patient cohort

We performed an electronic database search, showing that 1144 vestibular schwannomas were surgically treated in the authors’ institution between October 2003 and March 2017. Paraffin-embedded tissue samples of 1048 samples were available for further processing into tissue microarrays and microscopic analysis after immunohistochemical staining for COX2. Clinical data of all patients were collected, consisting of age, gender, prior regular medication with aspirin (daily dose of 100 mg or more) and other COX2-responsive medication, prior radiation therapy, confirmed diagnosis of neurofibromatosis type 2, tumor extension according to the Hannover grading scale [25].

### Tissue microarray and immunohistochemistry

**Fig. 1:**
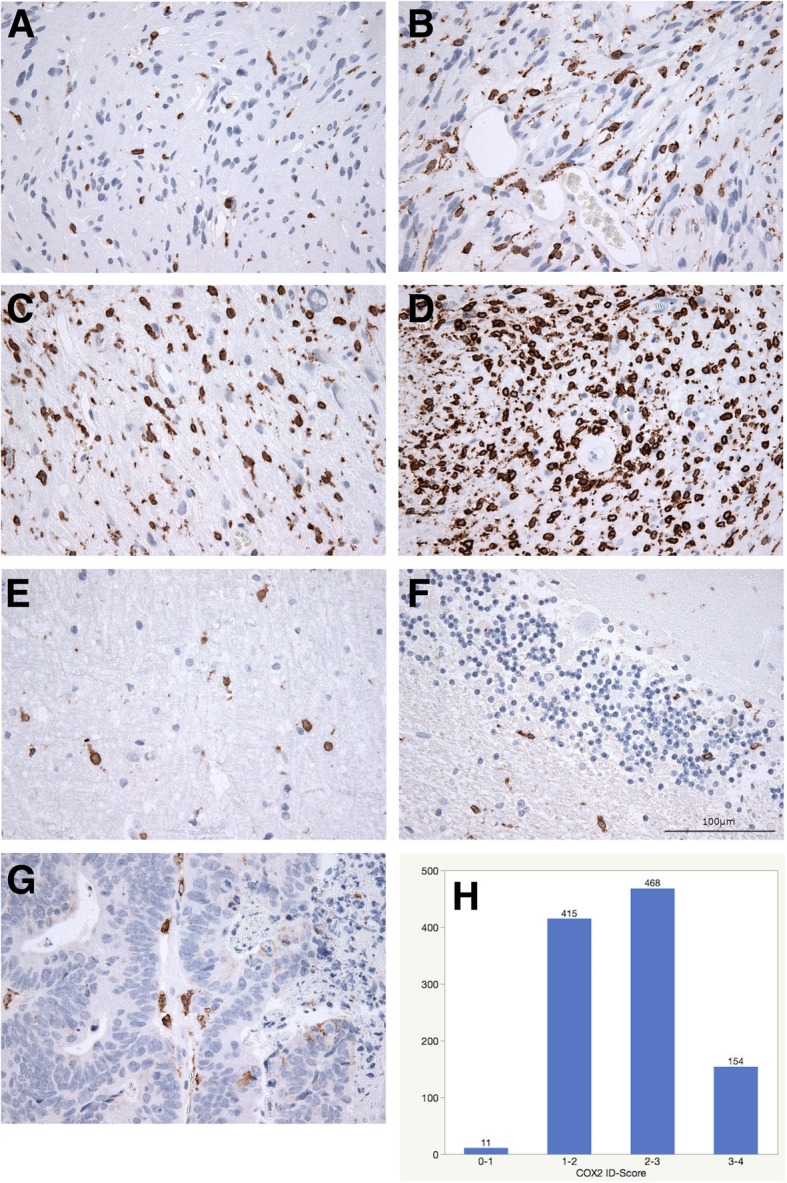
Intensity distribution score of COX2 immunohistochemistry: 5–25% immunopositivity = 1 (**a**); 25–50% = 2 (**b**); 50–75% = 3 (**c**); 75–100% = 4 (**d**); healthy cerebrum (**e**), cerebellum (**f**) and colorectal carcinoma metastasis (**g**) served as controls and showed only single immunopositive cells. The distribution of the ID-score is presented as a bar graph (**h**)

All samples were evaluated by two board-certified neuropathologists according to the current WHO classification of central nervous system tumors of 2016. After microscopic evaluation of H&E stains, eligible and representative areas of tumor samples were marked and tumor cylinder probes measuring 1 mm in diameter were extracted from the respective area of the corresponding paraffin embedded tumor tissue sample with a conventional tissue microarrayer (Beecher Instruments, Sun Prairie, Wisconsin, USA) and aligned on a recipient paraffin block. In most cases two sample cylinders were extracted from each tumor. Subsequently, 4 μm slices were produced from the TMA blocks with a microtome and dried at 80° Celsius for 15 min. Immunohistochemical staining was done with a Ventana BenchMark immunostainer (Ventana Medical Systems, Tucson, Arizona, USA). Pretreatment was performed with prediluted Cell Conditioning Solution CC1 (pH 8.5) for 40 min (MIB1) or CC2 (pH 6.0) for 32 min, followed by incubation with primary antibodies (MIB1 (DAKO, Hamburg, Germany) dilution 1:200, 42 °C, 42 min; COX2 (Biozol, Eching, Germany) dilution 1:800, room temperature, 32 min). Antibody incubation was followed by OptiView HQ Universal Linker for 12 min, incubation with OptiView HRP Multimer for 12 min. Both stains were finalized with counterstaining with one drop of hematoxylin for 4 min. Healthy human cerebral and cerebellar tissue and a sample of a colorectal carcinoma metastasis were used as controls and displayed positive cytoplasmic staining of microglia and macrophages respectively (see Fig. 1).

### Microscopic assessment and statistical methods

To determine the expression of COX2 microscopic assessment was done and a semiquantitative score was applied as delineated in Fig. 1. Immunopositivity of less than 5% was scored as “0”, between 5 and 25% as “1”, between 25 and 50% as “2”, between 50 and 75% as “3” and 75 to 100% as “4” (see Fig. 1a-d respectively). A differentiation of the COX2 staining intensity was not done since immunopositivity was usually quite strong and without much variation. For most tumors, 2 sample cylinders were available for analysis and a mean score was calculated accordingly. For multivariate nominal regression analysis, the cut off for COX2 expression was set at an ID-score of > 1 (more than 25% immunopositive tumor cells) since it was assumed to be the most easily evaluable cut off for daily neuropathology routine. For comparison, cut offs at > 2 and > 3 (50 and 75% immunopositivity, respectively) were also analyzed. MIB1 expression quantification was done with an automated evaluation of digitalized TMA slides. After digitalization of all TMA slides with a Mirax scanner (Zeiss, Göttingen, Germany), exported images were transferred to the Image J software (Version 1.51j8, NIH, Bethesda, MD, USA) with the plugins Bio-Formats (Release 5.4.1; Open Microscopy Environment, Madison, NJ, USA) and ImmunoRatio (Version 1.0c, Institute of Biomedical Technology, University of Tampere, Finland) to measure the percentual extent of nuclear staining.

Statistical analysis was done with JMP® (Cary, NC: SAS Institute Inc.; 1989). Classification and regression tree (CART) analysis, ANOVA and nominal logistic regression analysis were used and a significance level of α <  0.05 was applied.

## Results

### Demographic characteristics

Between October 2003 and March 2017, 1144 vestibular schwannomas were surgically treated in the authors’ institution. Ninety-six cases were excluded due to missing clinical data or tumor tissue that was not suitable for further processing and analysis, leaving 1048 vestibular schwannomas for final analysis.

The cohort included 115 schwannomas (11%) from NF2-patients and 37 tumors (4%) that received radiotherapy prior to resection. Regular preoperative ASA intake was recorded in 49 cases (5%). The mean age of the entire cohort was 46.5 years with NF2-patients being significantly younger (27.5 years, *p* = < 0.0001) and patients that reported regular ASA use significantly older (56.6 years, *p* = < 0.0001). Patients that were treated with radiotherapy prior to surgery or were re-operated did not a have a significant age difference. Specific information on the age distribution is listed in Table 1.

**Table 1:** Subgroup analysis of patient age

	Age in years	*p*-value (ANOVA)
Mean	46.5	–
Median	47.3	–
Range	7.1–79.1	–
Subgroups		
NF2 VS	27.5	< 0.0001*
Prior RT	50.2	0.1153
Recurrent VS	45.2	0.4658
Hannover Classification T3/4	46.5	0.9879
ASA intake	56.6	< 0.0001*

### COX2 immunohistochemistry

Overall uniformly strong cytoplasmic COX2 expression was seen in almost all tumor samples and showed a homogenous pattern (see Fig. 1). Part of the COX2-positive cells resembled intratumoral macrophages. Only 3 cases showed an ID-score of 0, meaning that 0–5% of the tumor cells were immunopositive. Eight tumors scored between zero and 1 (< 25% immunopositivity). The majority of the samples was ranging between 1 and 2 (25–50% immunopositivity, *n* = 415 39.6%) and 2 to 3 (50–75% immunopositivity, *n* = 468, 44.7%). One hundred and forty-four cases showed exceptional high ID-scores between 3 and 4 (75–100% immunopositivity, *n* = 144, 13.7%).

### Univariate ANOVA analysis

**Fig. 2:**
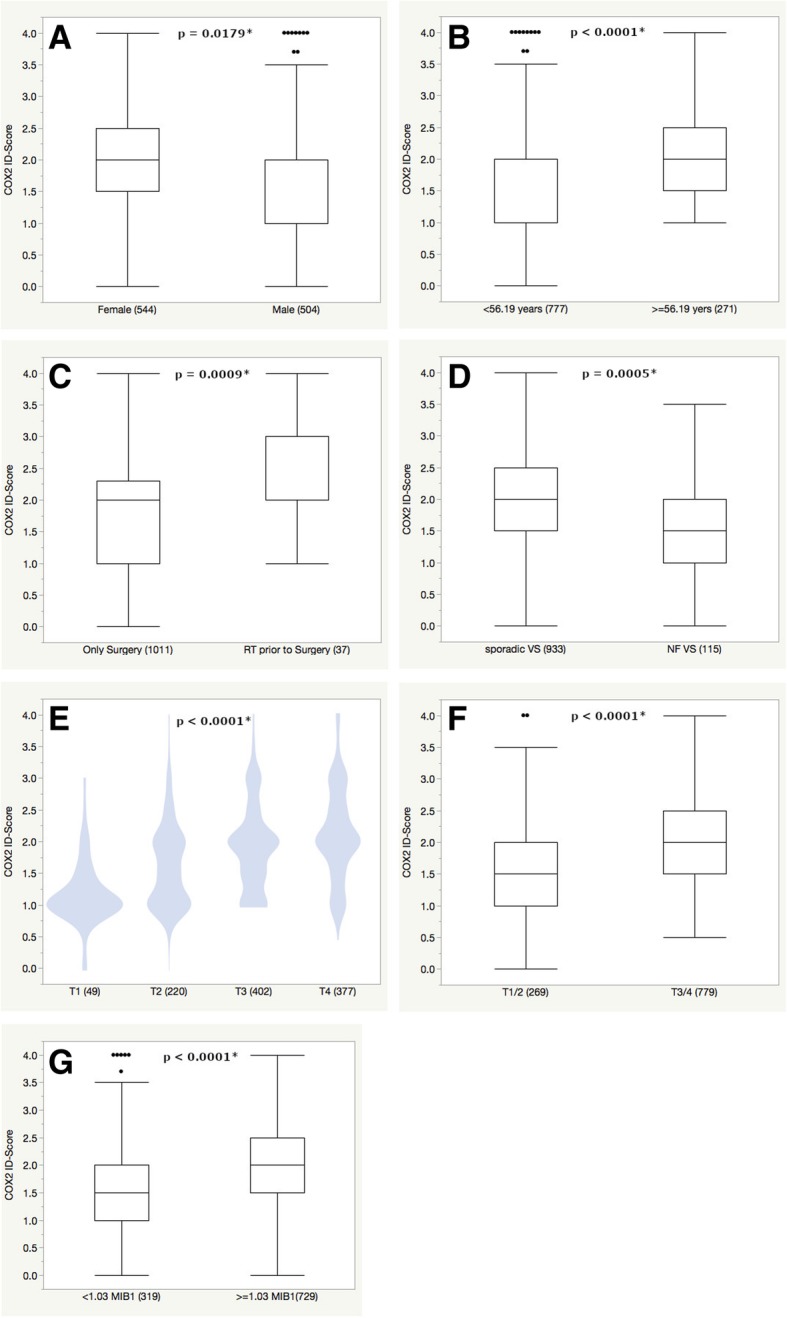
Significant differences of COX2 expression were observed for gender (**a**), age at the cut off 56.19 years (**b**), prior radiotherapy (**c**), NF2 (**d**), tumor expansion according to the Hannover grading scale (**e** and **f**) and expression of the proliferation marker MIB1 at the cut off 1.03% (**g**). Outliers are represented as single dots and were included in each analysis and respective *p*-values are shown at the top of each graph

Female patients had a significantly higher COX2 expression with an ID-score of 1.95 compared to male patients 1.84 (*p* = 0.0179). Vestibular schwannomas of NF2-patients showed a mean COX2 expression of 1.67 which was significantly lower compared to sporadic VS (ID-score 1.92, *p* = 0.0005, see Table 2 and Fig. 2). On the other hand, VS that were treated with radiotherapy prior to resection had a mean COX2 expression of 2.29 which was significantly higher compared to non-radiated tumors (ID-score 1.88, *p* = 0.0009, see Table 2 and Fig. 2).

**Table 2:** Univariate analysis of COX2 expression (ID-Score)

	n (%)	Mean (95%CI)	*p*-value (ANOVA)
Age (years)			
< 56.19	777 (74)	1.83 (1.78–1.89)	< 0.0001*
> =56.19	271 (26)	2.07 (1.99–2.16)	
MIB1 (% expression)			
< 1.03	319 (30)	1.68 (1.60–1.76)	< 0.0001*
> =1.03	729 (70)	1.99 (1.94–2.04)	
Gender			
Female	544 (52)	1.95 (1.89–2.01)	0.0179*
Male	504 (48)	1.84 (1.78–1.90)	
Primary/Recurrence			
Primary	992 (95)	1.89 (1.84–1.94)	0.7074
Recurrence	56 (5)	1.93 (1.74–2.13)	
Subtype			
Sporadic VS	933 (89)	1.92 (1.88–1.97)	0.0005*
NF2 VS	115 (11)	1.67 (1.54–1.81)	
Prior Treatment			
None	1011 (96)	1.88 (1.84–1.93)	0.0009*
Radiotherapy	37 (4)	2.29 (2.05–2.53)	
Hannover Classification			
T1	49 (5)	1.17 (0.98–1.37)	< 0.0001*
T2	220 (21)	1.62 (1.53–1.71)	
T3	402 (38)	1.92 (1.85–1.99)	
T4	377 (36)	2.12 (2.05–2.20)	
T1/2	269 (26)	1.54 (1.45–1.62)	< 0.0001*
T3/4	779 (74)	2.02 (1.97–2.07)	
Medication			
ASA	49 (5)	2.03 (1.82–2.23)	0.2046
No ASA	999 (95)	1.89 (1.84–1.94)	
NSAID	77 (7)	1.93 (1.76–2.09)	0.6887
No NSAID	971 (93)	1.89 (1.85–1.94)	
COX2-Inhibitor	2 (0.2)	2.25 (1.23–3.27)	0.4967
No COX-Inhibitor	1046 (99)	1.90 (1.85–1.94)	
Cortisol	27 (3)	2.02 (1.74–2.30)	0.3821
No Cortisol	1021 (97)	1.89 (1.85–1.94)	
Immunosuppressants (incl. cortisol)	32 (3)	2.00 (1.74–2.26)	0.4183
No immunosuppressants	1016 (97)	1.89 (1.85–1.94)

Tumor extension according to the Hannover classification [14] was also associated with significant differences in COX2 expression. Small tumors (T1) had the lowest expression with 1.17, with a subsequent increase over T2 (1.62) and T3 (1.92) up to a COX2 ID-Score of 2.12 for T4 vestibular schwannomas (*p* <  0.0001, see Fig. 2 and Table 2). Comparing T1 and T4 tumors directly, the COX2 expression almost doubled from 1.17 to 2.12.

**Fig. 3:**
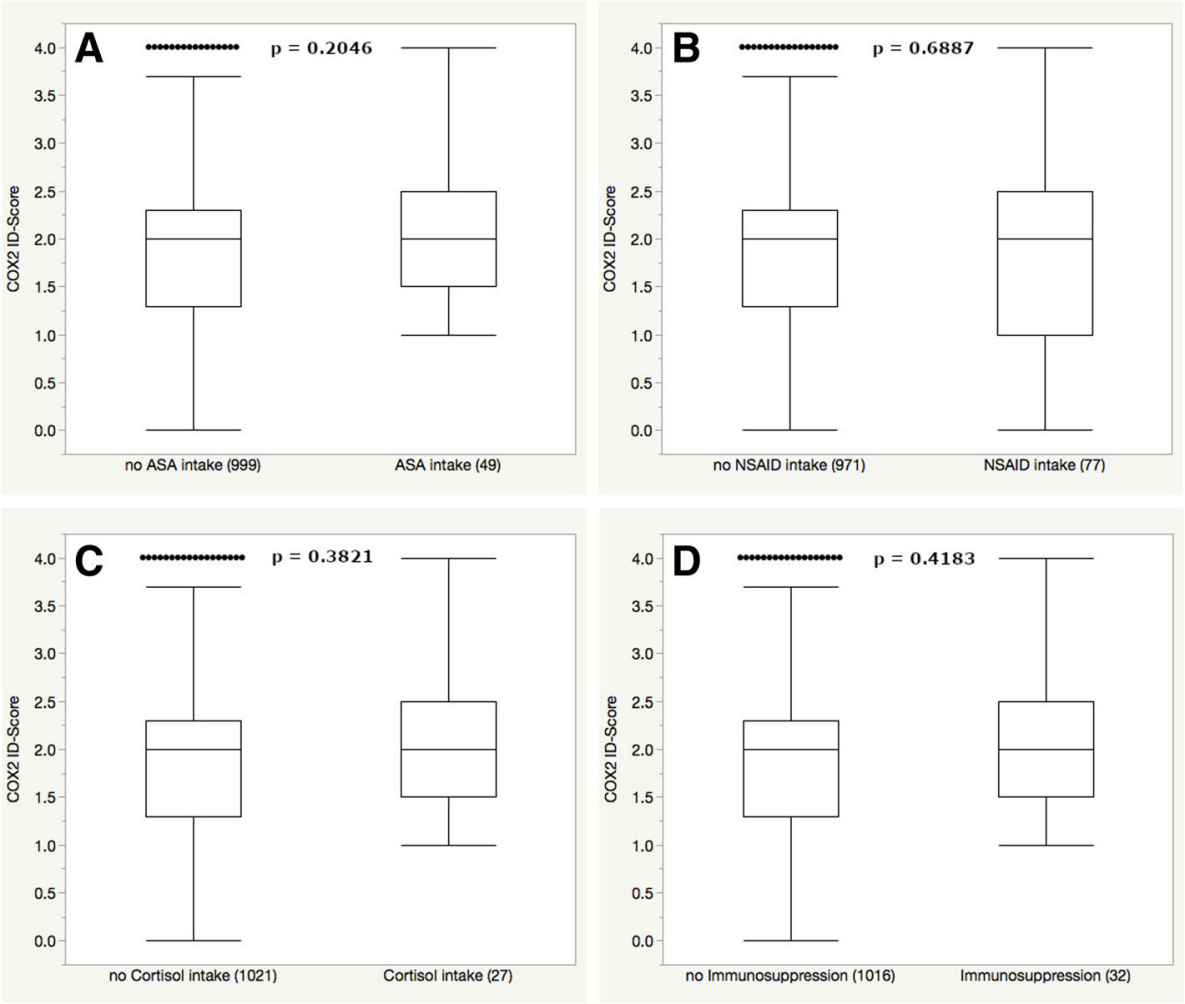
No significant difference in COX2 expression was observed for the intake of ASA (**a**), NSAIDs (**b**), glucocorticoids (**c**) or other immunosuppressants (**d**). Outliers are represented as single dots and were included in each analysis and respective *p*-values are shown at the top of each graph

Vestibular schwannomas of individuals that were on regular ASA, NSAIDs, glucocorticoids or other immunosuppressant agents did not show a difference in COX2 expression levels (see Table 2 and Fig. 3).

### Proliferation and COX2

As a marker for tumor proliferation, MIB1 expression was quantified for all evaluated tumor samples. It ranged from 0.0 to 4.75% immunopositivity of the nuclei of a tumor sample. For further analysis, COX2 expression was dichotomized at an ID-Score of 1, 2 and 3 and all three cut offs showed higher MIB1 expression rates within the group with the higher COX2 expression (*p* <  0.0001* at each cut off). A significantly higher MIB1 expression rate was seen in male patients (1.36 compared to 1.28%, *p* = 0.0092*). Patients with regular glucocorticoid medication also had higher MIB1 expression rates (1.53 compared to 1.31, *p* = 0.0284*). There was no significant difference in MIB1 expression for tumors of patients with regular ASA intake or other COX2-responsive mediations as well as NF2 patients, tumors receiving prior radiotherapy, tumor extension or patient age.

### Nominal logistic regression

We performed a multivariate analysis, including gender, ASA-intake, prior RT and presence of NF2, tumor extension together with patient age and MIB1 expression. A classification and regression tree (CART) analysis was applied to determine the optimal cut offs with the maximum difference in COX2 ID-score for MIB1 expression (> = 1.03%) and patient age (> = 56.19 years). For the nominal logistic regression, the cut off for COX2 expression was set at an ID-score of 1 (> 25% of immunopositive tumor cells, *n* = 791).

In contrast to the univariate ANOVA results, patient age and prior radiotherapy were no independent significant factors influencing COX2 expression, while ASA intake remained without significant impact as well (see Table 3).

**Table 3:** Multivariate nominal logistic regression analysis of COX2 expression (ID-Score)

	Odds Ratio (95%CI)	*p*-value (Prob > Chisq)
Female Gender	1.39 (1.02–1.90)	0.0355*
Age > = 56.19 years	1.12 (0.77–1.62)	0.5686
Prior Radiotherapy	2.55 (0.74–8.75)	0.1362
NF2 (vs. Sporadic VS)	0.45 (0.28–0.73)	0.0012*
MIB > =1.03	2.66 (1.94–3.67)	< 0.0001*
Hannover Classification		
T1-T2	3.72 (1.83–7.58)	0.0003*
T2-T3	2.65 (1.81–3.86)	< 0.0001*
T3–4	1.77 (1.19–2.63)	0.0046*
T1-T4	17.5 (8.46–36.1)	< 0.0001*
ASA intake	1.35 (0.63–2.90)	0.4349

With an odds ratio of 1.39 female gender was identified as an independent risk factor for increased COX2 expression. An immunopositivity of 1.03% or more of the tumor cells for the proliferation marker MIB1 was also associated with a higher rate of COX2 expression (OR 2.66 (1.94–3.67, *p* <  0.0001). Vestibular schwannomas of NF2 patients had significantly lower rates of COX2 expression (OR 0.45 (0.28–0.73), *p* = 0.0012). The extent of tumor extension according to the Hannover classification (T1 to T4) had the strongest association with increased COX2 expression. The highest odds ratio was seen between T1 and T2 with 3.72 (1.83–7.58, *p* = 0.0003) and subsequently decreased with higher categories. Between T2 and T3 the odds ratio was 2.65 (1.81–3.86, *p* < 0.0001) and further decreased to 1.77 (1.19–2.63, *p* = 0.0046) when comparing T3 and T4. Regarding all subclasses from T1 (tumor restricted to the internal acoustic meatus) to T4 (compression of the brain stem by the tumor mass) the odds ratio was 17.5 (95%CI 8.46–36.1, *p* < 0.0001).

The nominal regression was also performed with COX2 cut offs at 2 and 3 (50 and 75% immunopositivity, respectively). Tumor extension, NF2 and MIB > 1.03% remained independent significant factors (data not shown).

## Discussion

We evaluated the expression of COX2 in the yet largest cohort of 1048 vestibular schwannomas including 115 schwannomas of NF2 patients and compared the expression with demographic factors as well as tumor extension, MIB1 expression and the intake of COX2 responsive medication.

### ASA and COX2

We were able to demonstrate that there was no significant difference in COX2 expression in vestibular schwannomas with the intake of ASA, NSAIDs, glucocorticoids or other immunosuppressive medications. The association of tumor growth in sVS and inflammatory histopathological characteristics has been shown in a few studies. An increase of the expression of COX2 was observed in vestibular schwannoma tissue [12] and a vestibular schwannoma cell line [7]. Prostaglandin levels, a product of COX2, can be decreased with ASA and result in reduced cell proliferation compared to a normal Schwann cell culture [7]. A significant decrease in tumor progression after ASA administration was also recently demonstrated in a mouse model [26]. Additionally, de Vries et al. revealed an increased rate of CD163 positive macrophages in ten fast-growing sVS compared to a group of ten slow-growing sVS [6]. In a recent study, Carvalho and colleagues demonstrated a bidirectional regulation of COX2 expression in canine mammary tumor cells and CD68 positive macrophages, assuming a tolerogenic tumor microenvironment [3]. Overall, there is growing evidence to support the importance of inflammation in vestibular schwannoma growth.

The role of immunosuppression in NF2 has been explored and has gained increasing interest in regard to the development of possible targeted treatment strategies. Merlin, the product of the deleted gene in NF2, is known to have an inhibitory effect on the mammalian target of rapamycin complex 1 (mTORC1), leading to increased mTORC1 activity in NF2 associated tumors [16]. Growth inhibition by rapamycin, an immunosuppressant drug, was demonstrated using an in vitro cell model and a mouse model for NF2 [10] followed by a phase II trial, where everolimus was applied to 10 NF2 patients, which failed to reach the primary endpoint of tumor volume reduction [11]. However, the involvement of COX2 as a downstream target of mTORC1 has been described in tuberous sclerosis complex associated tumors and downregulation of COX2 showed antiproliferative effects [20].

Three large retrospective studies have shown contradicting results regarding the effect of ASA intake on radiographic tumor growth of sporadic VS. One study demonstrated a clear reduction of tumor growth by volumetric measurement in 347 sVS [17]. On the contrary Hunter et al. showed no effect of ASA intake on tumor growth in a series of 564 VS [13]. A most recent study assessed 437 cases and found no impact on ASA as well [21]. The Congress of Neurological Surgeons recently recommended the administration of aspirin for patients with vestibular schwannomas as an antiproliferative treatment [32].

Our large dataset shows that the expression of COX2 is not significantly altered in vestibular schwannomas by regular ASA intake, besides the established regulatory impact of ASA on the expression of COX2 [33, 34]. Interestingly, tumor tissue that was exposed to ASA treatment prior to surgery showed slightly higher expression rates but, as already mentioned, without statistical significance. The dosage of regular ASA intake can vary in other countries but the dosage for cardiovascular disease or prophylaxis of cardiovascular disease in Germany is 100 mg daily, which was the case for all 49 patients with regular ASA intake. Sporadic ASA intake due to headache was not considered as regular intake. The medication was given for a few months to several years prior to surgery. However, it is unknown how the dosage needs to be in vivo to exert a decrease of COX2 expression and a subsequent antiproliferative effect as observed in vitro [7]. It is unlikely that the dosage that is used to sustain an antiplatelet effect will also be suitable to have an impact on COX2, especially since the antiproliferative regulation seems to be on a transcriptional level and not via an acetylation of the enzyme COX2 [33, 34]. A prospective phase II trial that is currently recruiting patients for the investigation of aspirin efficacy regarding growth inhibition in vestibular schwannomas administers 325 mg twice daily (NCT03079999). This trial will very likely solve the question whether ASA should be recommended as an antiproliferative treatment option for VS patients. Nonetheless, our data does not support this principle.

It is necessary to stress, that our data showed that the expression of COX2 was significantly lower in NF2 patients, challenging the concept of ASA recommendation for this patient subgroup even more. However, it is unknown which level of COX2 expression can be regarded as possibly responsive to COX2 inhibition resulting in an antiproliferative effect.

### Proliferation and inflammation

As a marker for tumor proliferation, MIB1 expression was quantified unbiased by computerized image analysis for all evaluated tumor samples and was found to be significantly associated with higher COX2 expression, suggesting a link between COX2 expression and tumor proliferation, which was previously described in a retrospective analysis of thirty VS [12]. Now we can confirm a robust association in a large retrospective cohort.

Overall, the MIB1 index is usually quite low within this slow growing tumor entity, with only 4 cases with indices over 4% in this study cohort of 1048 cases. Some may argue that the tumor growth may not be properly represented by MIB1 indices in vestibular schwannoma. However, in a case series with 15 clinically aggressive vestibular schwannomas that all showed a radiological growth rate of > 15 mm/year, MIB1 expression was significantly increased compared to a control group [14]. It has also been described that the risk of regrowth after VS resection is associated with an increased MIB1 expression [9, 15].

It should be kept in mind that VS growth is not induced by the eukaryotic cell-cycle alone. There is growing evidence of inflammation as a significant factor in tumor growth and proliferation in VS [6, 19, 26, 29]. It remains unclear if the widely spread COX2 expression in almost all vestibular schwannomas of this cohort, is due to tumor growth or an expression of inflammation. Certainly, a higher MIB1 expression may also be the result of increasing inflammation and macrophage invasion which also have proliferative properties.

It is established that Inflammation plays an important role in cancer and there is evidence of a protective effect of NSAID intake in certain cancers [27, 30]. More specific COX2 expression was identified as a prognostic factor in a subset of breast cancer patients [5]. In urethral bladder cancer COX2 expression was shown to be over 28-fold increased when compared to normal urethral mucosa and is associated with tumor invasion and recurrence [1]. The observed differences in COX2 expression in this cohort are far from such an increase which is due to the methods applied but significant differences were found. This phenomenon needs further profound assessment but has the potential to reveal new therapeutic targets like tumor associated macrophages [2, 24]. However, in this cohort regular intake of ASA, NSAIDs, glucocorticoids or other immunosuppressants did not show a decreased level of COX2 or MIB1 expression suggesting that the commonly used ASA dosages have no beneficial effect. On the contrary MIB1 indices were significantly higher in tissue samples that underwent regular glucocorticoid treatment, a finding that cannot be explained at this point.

### Tumor extension

While the initial vestibular schwannoma is restricted to the internal acoustic meatus, tumor growth has to overcome the bony border of the canal, resulting in dilatation of the canal with further growth. It is possible that this step during tumor progression may differ from tumor growth into the cerebellopontine cistern where there is less mechanical resistance. Accordingly, the tumor microenvironment may also be different.

The odds ratio between T1 and T2 showed the highest rate for COX2 expression with a subsequent decrease of the odds ratio between T2/T3 and T3/T4. If this can be linked to the anatomical and mechanical aspects of the surrounding tissue during tumor growth is unclear. In this cohort there was no association between tumor extension and MIB1 expression. Suggestions have been made that the current tumor size is a factor influencing future tumor growth [21]. However, in the study by MacKeith and colleagues, growth was defined as 2 mm increase in diameter, which does not represent the true percentual increase of the tumor volume and is obsolete for tumor volume and growth assessment [21, 31].

### Limitations

Overall, the retrospective design is the major limitation of the study. Especially the retrospective collection of clinical data can be problematic due to incomplete documentation. However, the regular intake of aspirin was well documented since it is crucial to discontinue substances that can compromise platelet function and coagulation before intracranial surgery. In all 49 patients regular aspirin intake was prescribed due to diagnosed cardiovascular disease or increased risk profile for cardiovascular disease. Thus, the subpopulation of patients with aspirin use was older. Due to the retrospective nature of the study this selection bias can not be completely neutralized. However we have addressed this issue by incorporating the factor age into the multivariate analysis which showed robust results. Since the dosage of regular ASA intake can vary in other countries, the dosage for cardiovascular disease or prophylaxis of cardiovascular disease in Germany is 100 mg daily which was the case for all 49 patients with regular ASA intake. The medication was given for a few months to several years prior to surgery. Sporadic ASA intake due to headache was not considered as regular intake.

Additionally, retrospective studies on benign tumor tissue are always prone to a surgical selection bias. Large tumors are more likely to be treated surgically without further follow up scans. Therefore small, clinically stable or non-growing tumors that did not need surgical treatment are underrepresented in this cohort where tissue markers were assessed. This bias cannot be controlled. Overall, within this large cohort of 1048 cases there is still a significant number of small T1 and T2 tumors that underwent surgical resection.

The tissue microarray method has known limitations mostly due to the analysis of a small portion of the tumor that may not be representative. Therefore, we extracted two 1000 μm tissue cylinders of representative tumor tissue for each tumor and avoided areas with non-tumorous tissue (stroma, vessels, etc.). The amount of extracted tissue is sufficient since there is usually no tumor tissue heterogeneity in vestibular schwannomas except for Antoni A and B growth patterns.

The immunohistochemical staining of COX2 may not be the optimal method to measure the effect of ASA on COX2 in vestibular schwannoma tissue. However, it has been known for quite some time that ASA has a far-reaching regulatory impact by inhibiting the expression of COX2 [33, 34]. Thus, the immunohistochemical staining is adequate to measure the effect of ASA on COX2 levels.

## Conclusion

COX2 expression is associated with the proliferation marker MIB1 and tumor extension but is not altered by regular use of ASA, NSAIDs, glucocorticoids or immunosuppressants.

## Data Availability

The datasets used and analyzed during the current study are available from the corresponding author on reasonable request.

## Abbreviations

ASA: Acetylsalicylic acid
CART: Classification and regression tree
CD163: Cluster of differentiation 163
CD68: Cluster of differentiation 68
COX2: Cyclooxygenase 2
MIB1: Mindbomb e3 ubiquitin protein ligase 1
mTORc1: Mammalian Target of rapamycin complex 1
NF2: Neurofibromatosis 2
NIH-AARP: National institute of health – american association of retired persons
NSAIDs: Non-steroidal antiinflammatory drugs
sVS: Sporadic vestibular schwannoma
TMA: Tissue microaray
VEGF-A: Vascular endothelial growth factor a
VS: Vestibular schwannoma
